# Morphological and molecular characterization of *Eimeria purpureicephali* n. sp. (Apicomplexa:Eimeriidae) in a red-capped parrot (*Purpureicephalus spurius*, Kuhl, 1820) in Western Australia

**DOI:** 10.1016/j.ijppaw.2016.01.003

**Published:** 2016-01-12

**Authors:** Rongchang Yang, Belinda Brice, Una Ryan

**Affiliations:** a School of Veterinary and Life Sciences, Murdoch University, Murdoch, Western Australia, 6150, Australia; b Kanyana Wildlife Rehabilitation Centre, 120 Gilchrist Road, Lesmurdie, Western Australia 6076, Australia

**Keywords:** 18S rRNA, *E*. *purpureicephali* n. sp., Morphology, Genetic characterization, Cytochrome c oxidase subunit I (COI) gene, Phylogeny

## Abstract

A new *Eimeria* species is described from a red-capped parrot (*Purpureicephalus spurius*). Sporulated oocysts (n = 31) were spherical to subspherical, with a rough bilayered oocyst wall 0.8 μm thick. Oocysts measured 24.0 × 22.8 (20.4–26.4 × 18.3–25.9) μm, oocyst length/width ratio, 1.10. Oocyst residuum, polar granule and micropyle were absent. Sporocysts are elongate-ovoid, 11.0 × 7.3 (12.7–9.2 × 7.9–6.6) μm, sporocyst length/width ratio, 1.51 (1.33–1.71). The thin convex Stieda body and indistinct substieda bodies were present and the sporocyst residuum was composed of numerous small granules less than 1.0 μm in diameter dispersed randomly. Each sporocyst contained 2 sausage-shaped sporozoites in head-to-tail arrangement. The sporozoite nuclei were located centrally surrounded by refractile bodies. Molecular analysis was conducted at two loci; the 18S ribosomal RNA gene and the cytochrome c oxidase subunit I gene. At the18S locus, the new isolate shared 99.0% genetic similarity with *Eimeria dispersa* and *Eimeria innocua* from the turkey. At the cytochrome c oxidase subunit I gene locus, this new isolate was most closely related to *E*. *dispersa and E*. *innocua*, presented 99.0% and 98.0% genetic similarity, respectively. This new isolate and *E*. *dispersa* grouped together in the same clade. Based on the morphological and molecular data, this isolate is a new species of coccidian parasite, which is named *Eimeria purpureicephali* n. sp. after its host, the red-capped parrot (*Purpureicephalus spurius*).

## Highlights

• A new *Eimeria* species (*E*. *purpureicephali* n. sp.) in a red-capped parrot.
• Morphology study: distinct to other validated *Eimeria* species.
• Genetic study: 99% similarity with *E*. *dispersa* at 18S and COI loci.

## Graphical abstract

**Figure:**
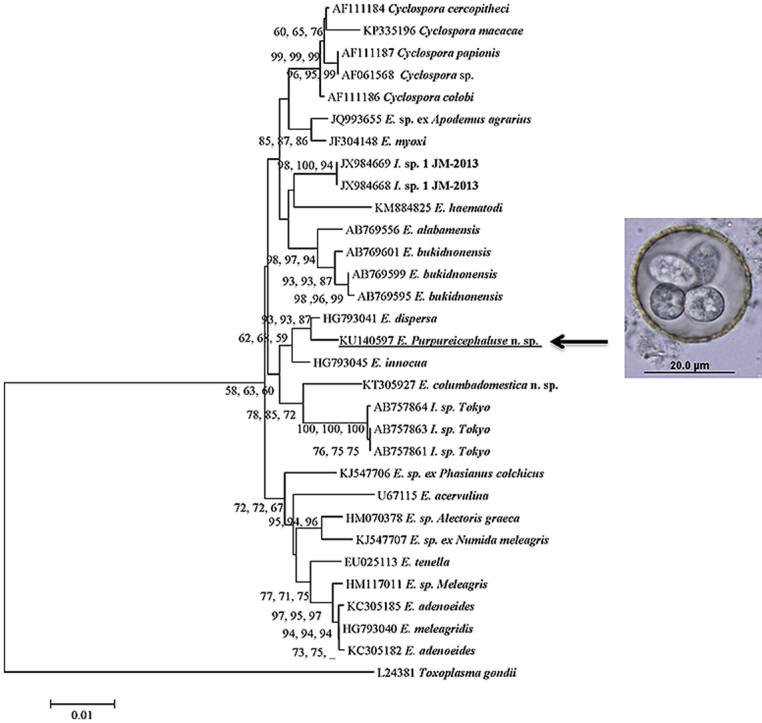

## 1 Introduction

The red-capped parrot (*Purpureicephalus spurius*), also called the pileated parakeet (Alderton, 2003) and king parrot locally in Western Australia (Lendon, 1973), is an Australian species of broad-tailed parrot which is related to the rosellas. The colourful red-capped parrot has a specialized long beak, which helps them to remove seeds from gumnuts of marri (*Eucalyptus calophylla*) as well as seeds from other eucalypts and native plants. These parrots live in eucalypt forests, woodlands, timbered watercourses, parks, orchards and gardens. Red-capped parrots are endemic to the south west of Western Australia. (Pizzey and Knight, 2007).

*Eimeria* (Coccidia: Eimeriidae), is a genus of apicomplexan parasites that includes various species and is known as the enteric monoxenous coccidian parasite. In birds, pathogenic *Eimeria* causes enteric disease and major economic losses in the global poultry industry (McDougald and Reid, 1997). *Eimeria* usually invade the intestinal tract, but some invade other organs, such as the liver and kidney. In recent years, more *Eimeria* species have been identified from free-range birds globally (Hofstatter and Guaraldo, 2011, Yang et al., 2014).

A total of four *Eimeria* species have been identified and recorded in the coccidian database (Duszynski et al., 2000) from the family Psittaciformes including *E*. *aratinga* (Upton and Wright, 1994), *Eimeria dunsingi* (Farr, 1960), *Eimeria haematodi* (Varghese, 1977) and *E*. *psittacina* (Gottschalk, 1972). Recently, a new *Eimeria* species, *Eimeria ararae* n. sp., from the blue-and-yellow macaw *Ara ararauna* (Linnaeus) in Brazil was added in the family Psittaciformes. With the exception of *E*. *haematodi*, which was molecularly characterized by Yang et al. (2015), the other four *Eimeria* species were identified by their oocyst morphological features only. To date there have been no reported cases of *Eimeria* species identified from the red-capped parrot (*Purpureicephalus spurius*, Kuhl, 1820). This is the first study to characterize *Eimeria purpureicephali* n. sp. in a red-capped parrot in Western Australia, using both morphological data and molecular techniques.

## 2 Materials and methods

### 2.1 Sample collection and examination

A juvenile red-capped parrot came into care at the Kanyana Wildlife Rehabilitation Centre (KWRC), Perth in November, 2014. On admission it was observed that this bird had labored breathing. Radiographs revealed multiple fractures of the keel bone and congestion of the air sacs. Reduced breast muscle mass was also noted. No clinical signs of coccidiosis were observed. Treatment was implemented but a decision was made to euthanize the bird a few days later. A faecal sample was taken soon after admission to look for evidence of avian gastric yeast (AGY). Microscopic examination of the faeces found no AGY in the sample, however unsporulated coccidian oocysts were seen.

Faecal flotation was conducted using a saturated sodium chloride and 50% sucrose (w/v) solution. A portion of faeces was placed in 2% (w/v) potassium dichromate solution (K_2_Cr_2_ O_7_), mixed well and poured into petri dishes to a depth of less than 1 cm and kept at room temperature in the dark to facilitate sporulation. Sporulated oocysts were observed using an Olympus DP71 digital micro-imaging camera and images were taken using Nomarski contrast with a 100× oil immersion objective. Faecal samples from another 23 red-capped parrots were screened for AGY (by wet mount) during the period January to December 2014. None of these 23 samples were found to be positive for coccidia.

A 3 axis hydraulic micromanipulator (MO-102, Nirashige, Japan) was used to isolate four separate single oocysts for DNA extraction and PCR.

### 2.2 DNA isolation

Oocyst DNA extraction was as described by Yang et al. (2014). Briefly, isolated single oocysts were placed on a slide and checked under the microscope (Olympus DP71 digital micro-imaging camera). Once the existence of a single oocyst on the cover slip was confirmed, photographs were recorded for morphological identification. The coverslip was then transferred into a PCR tube containing 10 μl of lysis buffer (0.005% SDS in TE solution). After a brief centrifugation, the tube was frozen in liquid nitrogen and thawed in a 95 °C water bath for four rounds to disrupt the oocyst wall. After the addition of 0.5 μl proteinase K (20 mM), the tube was incubated at 56 °C for 2 h and then at 95 °C for 15 min. The entire lysate from the single oocyst was used for three separate PCRs as described below.

### 2.3 PCR amplification and sequencing

A nested PCR with the primers EiGTF1 and EIGTR1 was used for the external amplification of the 18S rRNA gene. The expected PCR product was ∼1510 bp. The primers EiGTF2 and EiGTR2 (Yang et al., 2015) were used for the internal reaction.

A partial COI gene sequence (723 bp) was amplified using a nested PCR with the following primers COIF1 (Ogedengbe et al., 2011) and COXR1 (Dolnik et al., 2009) for the external reaction and COIF2 (Yang et al., 2013a) and COXR2 (Dolnik et al., 2009) for the internal reaction.

The amplicons from the second round PCRs were gel purified using an in house filter tip method as previously described (Yang et al., 2013b). All the PCR products were sequenced using forward and reverse primers in duplicate using amplicons from different PCR runs. An ABI Prism™ Dye Terminator Cycle Sequencing kit (Applied Biosystems, Foster City, California) was used for Sanger sequencing according to the manufacturer's instructions.

The results of the sequencing reactions were analysed and edited using FinchTV (Version 1.4), compared to existing *Eimeria* spp. 18S rRNA and COI sequences on GenBank using BLAST searches and aligned with reference genotypes from GenBank using Clustal W in BioEdit (V7.2.5).

### 2.4 Phylogenetic analysis

Phylogenetic trees were constructed for *Eimeria* spp. at the 18S, and COI loci with additional isolates from GenBank. Parsimony analyses were conducted using MEGA (Molecular Evolutionary Genetics Analysis software, version 6, Arizona State University, Tempe, Arizona, USA). Maximum likelihood (ML) and Neighbor-joining (NJ) analyses were conducted using Tamura-Nei based on the most appropriate model selection using ModelTest in MEGA 6. Bootstrap analyses were conducted using 1000 replicates to assess the reliability of inferred tree topologies.

## 3 Results

### 3.1 Species description

#### 3.1.1 Oocyst morphology

**Fig. 1:**
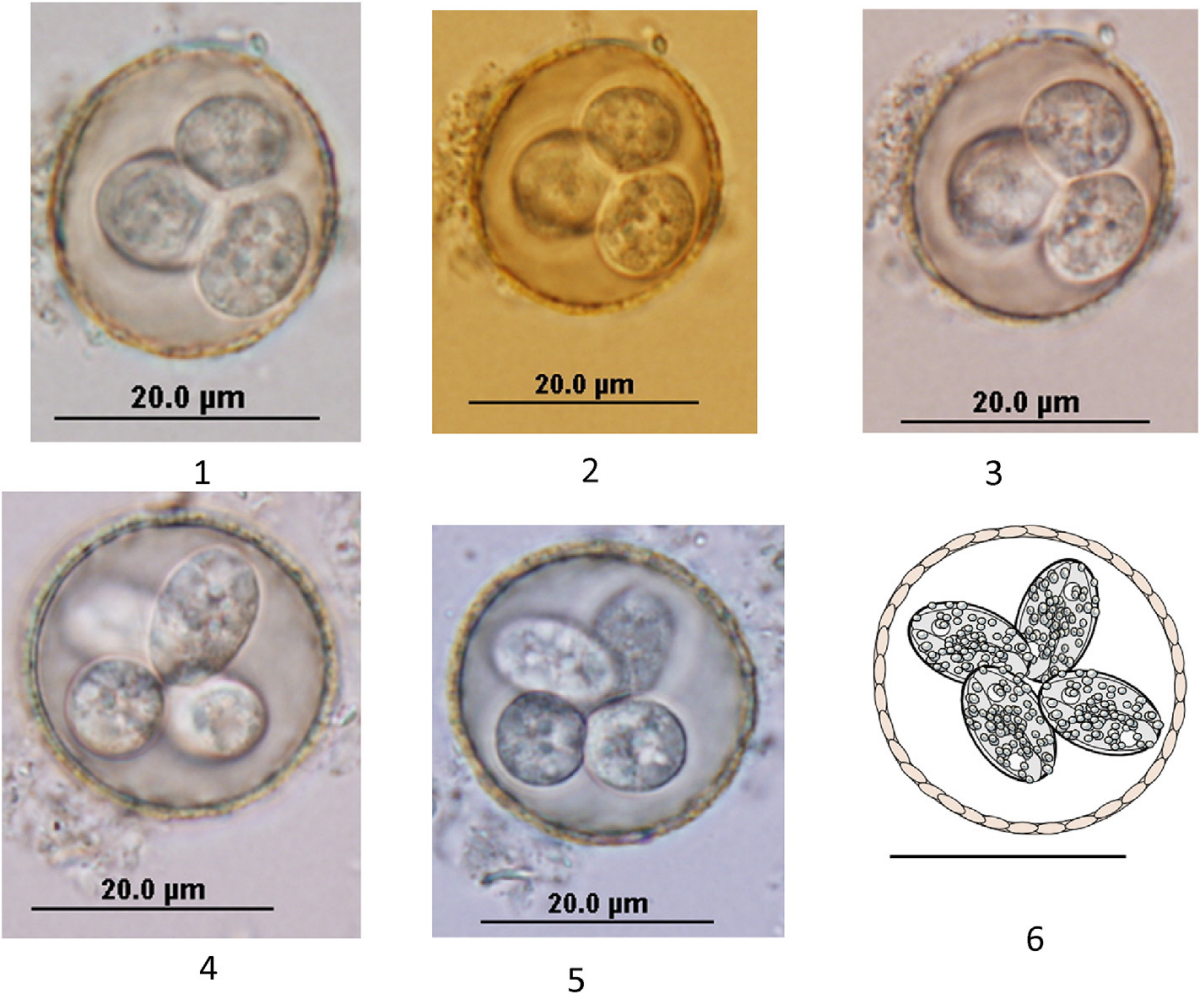
Nomarski interference-contrast photomicrographs of *E*. *purpureicephali* n. sp. oocysts showing spheroidal to subspheroidal sporocysts (scale bar = 20 μm) (1–5) and line drawing of the sporulated oocyst of *E*. *purpureicephali* n. sp. Scale bar = 20 μm (6).

Sporulated oocysts are subspherical, with a rough bilayered oocyst wall (0.8 μm thick). Oocysts measured 24.0 × 22.8 (20.4–26.4 × 18.3–25.9) μm, oocyst length/width (L/W) ratio, 1.10. Oocyst residuum, polar granule and the micropyle were absent. Sporocysts are elongate-ovoid, 11.0 × 7.3 (12.7–9.2 × 7.9–6.6) μm, sporocyst L/W ratio, 1.5 (1.3–1.7). A thin convex Stieda body and indistinct substieda bodies were present and the sporocyst residuum was composed of numerous small granules less than 1.0 μm in diameter dispersed randomly. Each sporocyst contained 2 sausage-shaped sporozoites in head-to-tail arrangement. The sporozoite nuclei were located centrally surrounded by refractile bodies (Fig. 1).

*Host*: Red-capped parrot (*Purpureicephalus spurius*: Psittaciformes).
*Locality*: Perth, Western Australia.
*Prevalence*: Unknown
*Other hosts*: Unknown.
*Prepatent period*: Unknown.
*Patent period*: Unknown.
*Site of infection*: Unknown.
*Sporulation time*: 72–96 h.

#### 3.1.2 Etymology

*Eimeria purpureicephali* n. sp. is named after its type host, the red-capped parrot (*Purpureicephalus spurius*).

#### 3.1.3 Material deposited

Oocysts in 10% formalin and oocyst phototypes were deposited in Western Australian Museum under the reference number WAMZ68782. DNA sequences have been deposited in GenBank under accession numbers KU140597 and KU140598 for the 18S and COI loci, respectively.

### 3.2 Phylogenetic analysis of *E*. *purpureicephali* n. sp. at the 18S locus

**Fig. 2:**
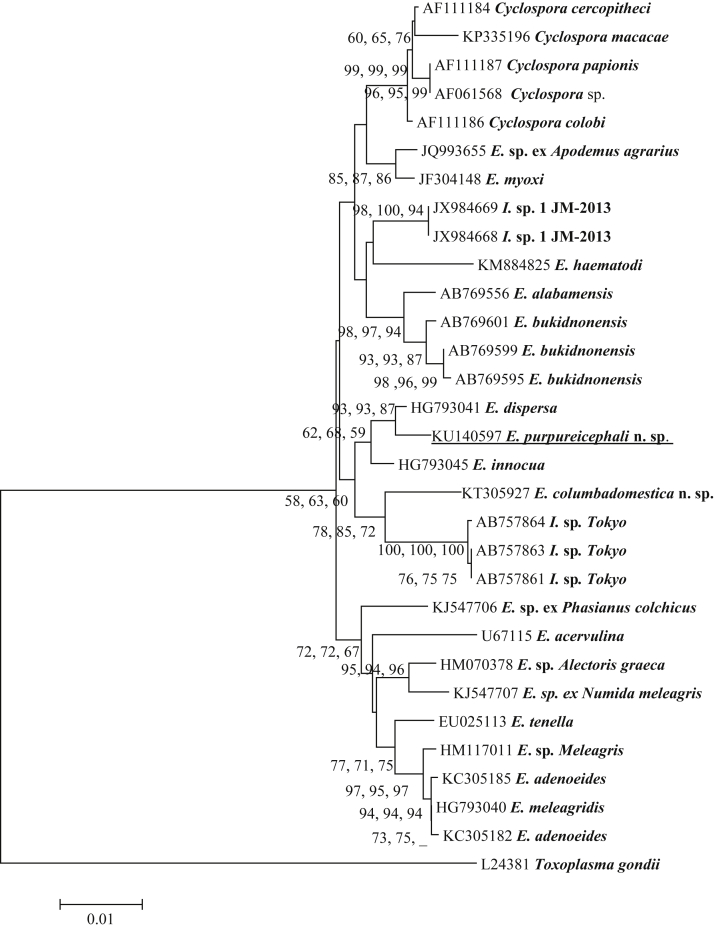
Evolutionary relationships of *E*. *purpureicephali* n. sp. inferred by distance analysis of 18S rRNA sequences (1229 bp). Percentage support (>50%) from 1000 pseudoreplicates from distance, ML and parsimony analysis, respectively, is indicated at the left of the support node (‘_’ = value was <50%).

A 1229 bp 18S rRNA PCR product of *E*. *purpureicephali* n. sp. was successfully amplified and sequenced. Analysis of three individual oocysts produced identical 18S rRNA sequences. Phylogenetic analyses of *E*. *purpureicephali* n. sp. at this locus using Parsimony, ML and NJ analyses produced similar results (Fig. 2, ML tree shown). *Eimeria purpureicephali* n. sp. grouped in a clade with *Eimeria dispersa* (HG793041) from a turkey in the Czech Republic and shared 99.0% genetic similarity (Fig. 2). It exhibited 98.0% genetic similarity to *Eimeria innocua* (HG793045), which was also identified from a turkey from the Czech Republic (Vrba and Pakandl, 2014). It shared 96.5% similarity with *E*. *haematodi* (KM884825) from a rainbow lorikeet (*Trichoglossus haematodus*: Psittaciformes) in Western Australia (Yang et al., 2015).

### 3.3 Phylogenetic analysis of *E*. *purpureicephali* n. sp. at the COI locus

**Fig. 3:**
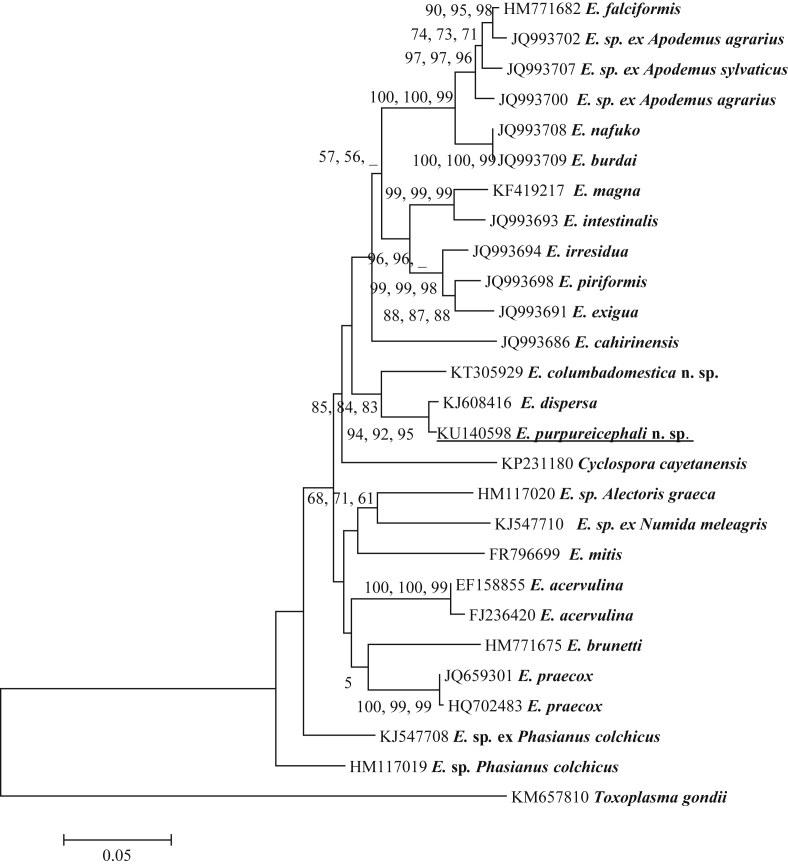
Evolutionary relationships of *E*. *purpureicephali* n. sp. inferred by distance analysis of COI sequences (670 bp). Percentage support (>50%) from 1000 pseudoreplicates from distance, ML and parsimony analysis, respectively, is indicated at the left of the support node (‘_’ = value was <50%).

Phylogenetic analysis of the 670 bp COI sequence placed *E*. *purpureicephali* in a clade with *E*. *dispersa* (KJ608416) (99.0% similarity), a turkey-derived *Eimeria* (Fig. 3). It also exhibited 94.8% similarity with *Eimeria columbadomestica* n. sp. (KT305929), which was identified from a domestic pigeon. These three sequences grouped in a separate clade from other *Eimeria* species (Fig. 3). As only short COI sequences (220–311 bp) were available from *E*. *haematodi* and other *Eimeria* species, they were not included in the phylogenetic analysis. Pair-wise comparison of the partial COI gene sequence (311bp) between *E*. *purpureicephali n*. *sp*. and *E*. *haematodi* was conducted and they shared 93.6% genetic similarity.

## 4 Discussion

Sporulated oocysts of *E*. *purpureicephali* n. sp. are morphologically distinct from other characterized *Eimeria* species and did not match any other existing documented *Eimeria* species (n = 11) from Psittaciformes (http://biology.unm.edu/biology/coccidia/Psittaciformes.html (Accessed on 28th Dec. 2015) (Table 1). The dimensions of the oocysts from *E*. *purpureicephali* n. sp. were smaller than those of the *Eimeria* species from the hosts in the family Psittaciformes (Table 1).

Phylogenetic analysis of 18S rRNA and COI sequences based on ML, NJ and Parsimony analyses produced similar results and placed *E*. *purpureicephali* n. sp. in a clade with *E*. *dispersa* (both at 99.0% genetic similarity). The genetic similarity between *E*. *dispersa* and *E*. *haematodi* was 95.5% at the 18S rRNA locus based on 1235 bp of common sequence and 93.6% at the COI locus based on 311 bp of common sequence.

As *E*. *purpureicephali* n. sp. exhibited a high genetic similarity with *E*. *dispersa*, at the 18S rRNA and COI loci, oocyst morphological features between these two species were compared in Table 1 and the dimensions of the oocysts and sporocysts were different between the two species. For example, oocysts of *E*. *dispersa* (22.4 × 18.0 μm) are smaller than those of *E*. *purpureicephali* n. sp (24.0 × 22.8 μm) (Table 1). *Eimeria dispersa* was originally described from a turkey (Tyzzer, 1929) and based on cross-transfection studies, it infected chickens and other gallinacerous birds (Tyzzer, 1929, Hansen, 1974, Doran, 1978). To date, there has been no report about *E*. *dispersa* in parrots. Therefore, *E*. *purpureicephali* n. sp from this study is different from *E*. *dispersa* even though they shared 99.0% genetic similarity at the 18S rRNA and COI loci. This is yet another example demonstrating the importance of utilising both traditional morphological data and molecular tools for analysis of coccidian taxonomy, as morphological or molecular data used on their own may lead to incorrect species identification. As more sequence data from avian-derived *Eimeria* species becomes available on GenBank, particularly from passerine birds, a more robust molecular taxonomy will be developed.

The incidence rate of coccidia in red-capped parrots in the Perth area was 4.2% (1/24). The true incidence rate is probably higher as the faecal samples were only screened by wet mount and not by faecal float. AGY was seen in 20.8% (5/24) of the samples tested. Two of the five red-capped parrots that were positive for AGY, were also exhibiting clinical signs of Psittacine beak and feather disease.

In conclusion, this is the first report of the morphological and molecular characterization of an *Eimeria* species in the red-capped parrot from Australia. Further studies of the life cycle and pathogenicity of *E*. *purpureicephali* n. sp as well as multi-locus sequencing are necessary to further characterize this *Eimeria* species.

## Conflicts of interest

The authors declare that there is no conflict of interest.

## Figures and Tables

**Table 1:** Comparative morphology of *E*. *purpureicephali* n. sp. from red capped parrot in Perth, WA with other species recorded from psittacine birds and *E*. *dispersa*.

Species	Hosts	References	Oocysts					Sporocysts					
Shape	Size (μm)	Shape index	Wall	Oocyst Residuum	Polar granule	Shape	Size (μm)	Stieda body	Substieda body	Residuum
*E. aestivae*	Blue-fronted parrot (*Amazona aestiva*)	Hofstatter and Guaraldo, 2011	Ovoidal	36.8 × 23.7	1.55	Bi-layered	Absent	Single rounded polar granule	Ovoidal	19.8 × 9.3	Present	Present	Present
*E. amazonae*	Yellow-crowned parrot (*Amazona ochrocephala*)	Hofstatter and Kawazoe, 2011	Ellipsoidal	48.9 × 36.2	1.35	Bi-layered	Absent	Single rounded polar granule	Ellipsoidal	22.2 × 11.9	Present	Present	Present
*E. ararae*	Blue-fronted parrot (*Amazona aestiva*)	do Bomfim Lopes et al., 2014	Ovoidal	28.7 × 20.2	1.42	Bi-layered	Absent	Present, 2 to 4 granules	Elongate-ovoidal	17.0 × 8.3	Present	Absent	Granular
*E. aratinga*	Orange-fronted conure (*Eupsittula canicularis*)	Upton and Wright, 1994	Ellipsoidal	35.0 × 25.9	1.35	Bi-layered	Absent	Present and fragmented	Elongate-ovoidal	17.0 × 8.3	Present	Present	Present
*E. dispersa*	Quail (*Colinus virginianus*)	Hansen, 1974	Subspherical to ovoid	22.4 × 18.5	1.21	Bi-layered	Absent	Single polar granule	Ellipsoidal	14.1 × 6.7	Present	Present	Granular
*E. dunsingi*	Musk lorikeet (*Glossopsitta concinna*)	Gartrell et al., 2000	ovoid	35.8 × 23.0	1.56	Bi-layered	ABSENT	Single polar granule	Ovoid to pyriform	11.4 × 8.5		Absent	Granular
*E. haematodi*	Rainbow lorikeet (PNG) (*Trichoglossus haematodus*)	Varghese, 1977	Ovoid to slightly piriform	32.3 × 27.6	1.17	Bi-layered	Present	Absent	Ellipsoidal	13.3 × 8.4	Present	Absent	Granular
*E. haematodi*	Rainbow lorikeet (WA) (*Trichoglossus haematodus*)	Yang et al., 2015a	Ovoid to slightly piriform	33.3 × 28.1	1.19	Bi-layered	Present	Absent	Spherical to ovoid	12.2 × 8.3	Present	Absent	Granular
*E. ochrocephalae*	Yellow-crowned parrot (Amazona ochrocephala)	Hofstatter and Guaraldo, 2011	Ellipsoidal	43.8 × 27.7	1.55	Bi-layered	Absent	Single polar granule, rounded	Ovoidal	20.6×	Present	Present	Granular
*E. purpureicephali n. sp.*	Red-capped parrot (*Purpureicephalus spurius*)	Present study	Spherical to subspherical	24.0 × 22.8	1.1	Bi-layered	Absent	Absent	Elongate-ovoid	11.0 × 7.3	Present	Absent	Granular
